# Predictors for resectability and survival in locally advanced pancreatic cancer after gemcitabine-based neoadjuvant therapy

**DOI:** 10.1186/1471-2482-14-72

**Published:** 2014-09-25

**Authors:** Ying-Jui Chao, Edgar D Sy, Hui-Ping Hsu, Yan-Shen Shan

**Affiliations:** 1 Division of General Surgery, Department of Surgery, National Cheng Kung University Hospital, Tainan, Taiwan; 2 Institute of Clinical Medicine, College of Medicine, National Cheng Kung University, Tainan, Taiwan

**Keywords:** Locally advanced pancreatic cancer (LAPC), Neoadjuvant therapy, Concurrent chemoradiation therapy (CCRT)

## Abstract

**Background:**

To evaluate the predictors for resectability and survival of patients with locally advanced pancreatic cancer (LAPC) treated with gemcitabine-based neoadjuvant therapy (GBNAT).

**Methods:**

Between May 2003 and Dec 2009, 41 tissue-proved LAPC were treated with GBNAT. The location of pancreatic cancer in the head, body and tail was 17, 18 and 6 patients respectively. The treatment response was evaluated by RECIST criteria. Surgical exploration was based on the response and the clear plan between tumor and celiac artery/superior mesentery artery. Kaplan–Meier analysis and Cox Model were used to calculate the resectability and survival rates.

**Results:**

Finally, 25 patients received chemotherapy (CT) and 16 patients received concurrent chemoradiation therapy (CRT). The response rate was 51% (21 patients), 2 CR (1 in CT and 1 in CRT) and 19 PR (10 in CT and 9 in CRT). 20 patients (48.8%) were assessed as surgically resectable, in which 17 (41.5%) underwent successful resection with a 17.6% positive-margin rate and 3 failed explorations were pancreatic head cancer for dense adhesion. Two pancreatic neck cancer turned fibrosis only. Patients with surgical intervention had significant actuarial overall survival. Tumor location and post-GBNAT CA199 < 152 were predictors for resectability. Post-GBNAT CA-199 < 152 and post-GBNAT CA-125 < 32.8 were predictors for longer disease progression-free survival. Pre-GBNAT CA-199 < 294, post-GBNAT CA-125 < 32.8, and post-op CEA < 6 were predictors for longer overall survival.

**Conclusion:**

Tumor location and post-GBNAT CA199 < 152 are predictors for resectability while pre-GBNAT CA-199 < 294, post-GBNAT CA-125 < 32.8, post-GBNAT CA-199 < 152 and post-op CEA < 6 are survival predictors in LAPC patients with GBNAT.

## Background

Pancreatic cancer is the most formidable malignancy with annual mortality nearly equal to its annual incidence and the 5-year survival rate was less than 5%. Surgical resection is the only potentially curative treatment. However, less than 20% cases are eligible for resection at presentation with a 5-year survival rate of 20% [1]. Locally advanced pancreatic cancer (LAPC) is defined as surgically unresectable pancreatic cancer involving the celiac artery or the superior mesentery artery without evidence of distant metastasis [2]. It accounts for 26% of newly diagnosed pancreatic cancer with a 5-year survival rate of 8.7% [3]. With amelioration of the resectability of LAPC, the overall survival of pancreatic cancer can be improved.

Several randomized trials have been performed to increase the resectability of LAPC. The 5-fluorouracil (5-FU) based chemoradiotherapy has been supported as the most acceptable treatment [4-6]. In the recent decade, gemcitabine has been considered as the standard agent for advanced pancreatic cancer and also act as a radiosensitizer during radiotherapy [7-9]. In year 2003, an algorithm for management of LAPC with intends of improving the survival rate and quality of life of patients was set up using gemcitabine-based neoadjuvant therapy in National Cheng Kung University Hospital. In this study, the effect on surgical resection, survival rate and predictors for resectability of patients with LAPC after neoadjuvant therapy with gemcitabine-based chemotherapy or gemcitabine-based concurrent chemoradiation therapy is presented.

## Methods

### Patients and treatments

From January 2003 to Dec 2009, patients with LAPC were enrolled for gemcitabine-based chemotherapy or gemcitabine-based concurrent chemoradiation therapy. The diagnosis of LAPC was based on thin sliced enhanced multi-detected computed tomography (MDCT) [10] with inclusion criteria of 1) abutment or encasement of celiac artery or superior mesentery artery (cT4) [2]; 2) the involvement of portal vein at the confluence superior mesentery vein and splenic vein [11]; 3) severe extra-pancreatic soft tissue involvement. All patients underwent CT-guide core-biopsy and proved to have pancreatic ductal adenocarcinoma. Patients, who had previous surgical exploration with proved pancreatic ductal adenocarcinoma was also enrolled. The treatment plan was based on the algorithm treatment of LAPC in the National Cheng Kung University Hospital. This study was approved by the Institutioal Review Board of National Cheng Kung University Hospital, ER-98-023. The informed consent for participation in the study was obtained from participants.

Three GBNAT were used for LAPC patients: 1) the first regimen was institutional phase II trial of chemotherapy (CT). The regimen was combined intravenous infusion of gemcitabine 1000 mg/m^2^ for 100 minutes and oxaliplatin 70 mg/m^2^ for 2 hours on day 1, and fluorouracil (5-FU) 1000 mg/m^2^ for 24 hours on day 2, and followed by oral thalidomide 100 mg per day after intravenous infusion therapy every 2 week for 6 cycles. 2) the second regimen was intravenous infusion of gemcitabine 1000 mg/m^2^ for 100 minutes and oxaliplatin 70 mg/m^2^ for 2 hours on day 1, and fluorouracil (5-FU) 1000 mg/m^2^ for 24 hours on day 2and followed by oral Sunitinib 12.5 mg per day after intravenous infusion therapy, every 2 week for 6 cycle. 3) the third regimen was gemcitabine-based concurrent chemoradiation therapy (CRT). In this regimen, patients received three-dimensional conformal radiotherapy at the target area, pancreatic lesion and nodal area, with a total dose of 50.4 Gy (1.8 Gy/day). The chemotherapy included concurrent 30-minute intravenous infusion of gemcitabine at a dose of 400 mg/m^2^ every week during radiation and 60-minute intravenous infusion of gemcitabine at a dose of 1000 mg/m ^2^ for three continuous weeks after radiation. The choice of gemcitabine-based CT or gemcitabine-based CRT was dependent on patient’s preference following explanation of the side effects of different regimens.

### Assessment of response and indications for operation

The response to treatment after GBNAT was restaged by MDCT scan of the abdomen at 8 weeks routinely. The response of downing size of the tumor was determined by the RECIST criteria. Surgical exploration was done in patients with evidence of downsizing of the tumor mass and the clear plan between tumor and celiac artery/superior mesentery artery after treatment. In patients with a stable disease, reevaluation of tumor response was performed at 12 weeks after GBNAT, and surgical exploration is performed only if there is evidence of downsizing of the mass or stable disease without increasing tumor markers. Tumor markers; CA199, CA125, and CEA were determined at pre-GBNAT, post-GBNAT and 1 months post-resection. During exploration, pancreatic resection (conventional Whipple’s procedure, pylorus-preserving pancreaticoduodenectomy, central pancreatectomy or distal pancreatectomy) with regional lymphadenectomy was determined by surgeon according to preoperative evaluation from MDCT, patients’ co-morbidities, and preoperative nutrition status. Pathologic stage was defined according to the American International Union against Cancer (AJCC) [2]. Resection margin were examined for defined radicality (including proximal and peripancreatic margins). Postoperative follow-up included routine chest X-ray, and MDCT of the abdomen every 3 months and every 6 months during the first and second postoperative year respectively or if patients had suspicious sign of recurrence. Routine bone scan was done every 6 months or when suspicious symptom of bone metastasis is present. Tumor markers were evaluated every 3 months during the first two postoperative years. Additional tests, cytologic examination of ascites and pleural effusion and brain CT, were performed when a recurrence or distant metastasis was suspected. In those patients with disease progression or distant metastasis, further chemotherapy, pain control, or best supportive care were arranged according to patient’s clinical condition based on our LAPC management algorithm.

### Statistical analysis

Continuous variables were expressed as mean ± S.D. and compared with a two-tailed *t-test*. Categoric variables were compared with Fisher’s exact test. Univariate and multivariate analyses for predictors of resectability rate were performed using Cox stepwise regression. Univariatee analysis for survival was calculated as the interval from registration until death using Kaplan-Meier method and the difference in survival between groups was compared using log-rank test. A univariate p ≤ 0.05 in each of these analyses were considered for entry into multivariate analyses Cox model calculation. Results were considered significant for value of p ≤ 0.05. All analyses were performed using SPSS statistical software (version 13^th^, SPSS, Inc., Chicago, IL).

## Results

**Figure 1:**
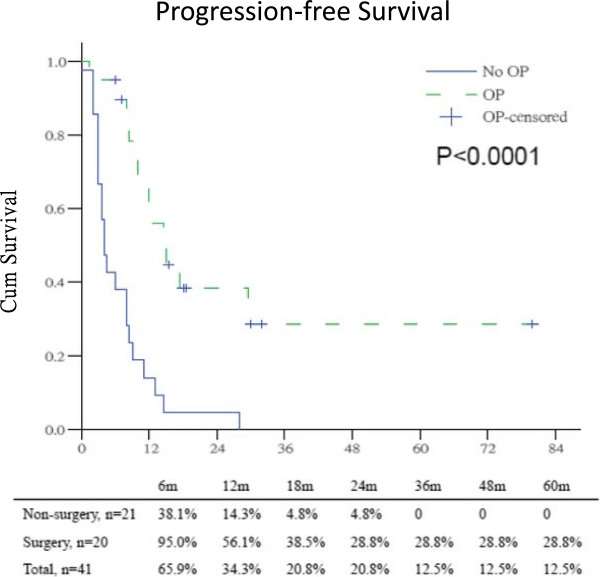
Progression-free survival between surgery and non-surgery patients in LAPC after gemcitabine-based neoadjuvant therapy.

**Figure 2:**
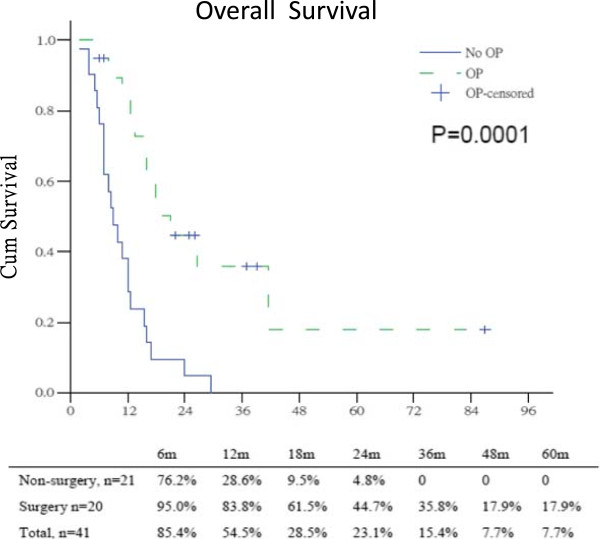
Overall survival between surgery and non-surgery patients in LAPC after gemcitabine-based neoadjuvant therapy.

Between May 2003 and Dec 2009, 41 tissue-proved LAPC patients, 27 male and 14 female, with mean age of 63.5 years were treated with GBNAT. There were 25 patients received gemcitabine-based CT and 16 patients received gemcitabine-based CRT in the National Cheng Kung University Hospital. 9 patients had previous failed surgical resection before enrollment and 32 patients were naïve patients with MDCT unresectable LAPC. The location of LAPC in the head, body and tail was 17, 18 and 6 patients respectively. The pre-GBNAT serum of tumor markers level were CEA 24.9 ± 28.6, CA199 1641 ± 3697, and CA125 51.1 ± 51.9. After treatment, the response rate was 51.2%, 2 CR and 19 PR. There were 10 PR and 1 CR in the 25 (44%) patients received gemcitabine-based CT, and 9 PR and 1 CR in the 16 (62.5%) patients received gemcitabine-based CRT. There were 20 patients received exploration by response rate and MDCT evaluation but 17 patients had successful tumor resection (14 R0, 2 R1 and 1 R2), in which 6, 5 and 6 patients received Whipple’s operation, central pancreatectomy, and distal pancreatectomy respectively. In these resectable cases, two pancreatic neck cancer patients received central pancreatectomy and pathology revealed fibrotic change only. The harvested and positive node number was lower. The positive surgical margin rate was 17.6% (3 of 17 patients). Three patients had failed surgical resection due to severe dense adhesion around the retroperitoneal region and SMA/SMV area. Those patients with R2 resection and failed surgical resection were all pancreatic head cancer with uncinate process extension (Table 1).After GBNAT, the level of tumor markers CEA, CA199 and CA125 were 20.3 ± 51.2, 1820 ± 4780, and 82.3 ± 135.5 respectively. The median progression-free survival was 9.0 (18.4 ± 3.0) months, patients with surgical exploration had significant longer progression-free survival than those without surgical exploration; 15.0 (32.1 ± 6.0) months versus 4.0 (6.7 ± 2.0) months. The 5-year progression-free survival rate was 12.5%, 28.5% in those patients with surgical exploration versus 0% in those patients without surgical exploration. In those patients only had 4.8% 2-year progression-free survival rate, P < 0.0001 (Figure 1). The median overall survival was 12.5 (20.8 ± 4.0) months, patients with surgical exploration had longer overall survival than those without surgical exploration; 21.0 (33.1 ± 7.0) months versus 9.0 (10.5 ± 2.0) months. The 5-year overall survival rate was 7.7%, 17.9% in those patients with surgical exploration compared to 4.8% in those patients without surgical exploration, P = 0.0001 (Figure 2). The progression-free survival and overall survival of LAPC with surgical exploration after GBNAT were similar to those of patients with initial primary resectable pancreatic cancer.

**Table 1:** Demographics of patients with locally advanced pancreatic cancer received gemcitabine-based neoadjuvant therapy (GBNAT) and surgical resection

**Characteristics**	**Number of patients, n = 41**
Sex (M:F)	27:14
Age (years), mean (range)	63.5 (39–80)
Location: Head: body: tail	17:18: 6
Naïve: failed exploration	32:9
Chemotherapy(CT)^#^: chemoradiation therapy (CRT)*	25:16
Response rate	21 (51.2%)
CR: PR	2:19
Surgery: non-surgery	20:21
Successful resection	17 (41.5%)
Surgical procedure	
Whipple: central pancreatectomy: distal pancreatectomy	6: 5: 6
Surgical margin	
R0: R1: R2	14: 2:1
Pre-GBNAT tumor marker level	
CEA	24.9 ± 28.6
CA199	1641 ± 3697
CA125	51.1 ± 51.9
Post-GBNAT tumor marker level	
CEA	20.3 ± 51.2
CA199	1820 ± 4780
CA125	82.3 ± 135.5
Progression-free survival, median (mean ± SD), months	9.0 (18.4 ± 3.0)
Surgery vs non-surgery	15.0 (32.1 ± 6.0) vs 4.0 (6.7 ± 2.0)
Overall survival, median (mean ± SD), months	12.5 (20.8 ± 4.0)
Surgery vs non-surgery	21.0 (33.1 ± 7.0) vs 9.0 (10.5 ± 2.0)

^#^17 patients received phase I/II GOFT, 8 patients with GOFS, *7 patients received CCRT Tainan program, 9 patients received gemcitabine induction chemotherapy and reduced dose gemcitabine with R/T.

There were 20 patients received planned surgical exploration after evaluation. However, only 17 patients received successful resection (Table 1). Comparison of patients without exploration or failed exploration, tumor location (tail vs head and body), post-GBNAT serum CA199 (<152 vs ≥152), a decrease of CA199 and CA125 before surgery (post-GBNAT) was significant predictors for resectability in the univariate analysis. However, after using multivariate analysis, tumor location (OR: 50, CI: 1.218~ > 100. P = 0.039) and post-GBNAT CA199 < 152 (OR: 14.686, CI: 1.114 ~ 193.6, P = 0.041) were significant predictors for resection after GBNAT (Table 2).

**Table 2:** Univariate and multivariate analysis of parameters associated with resectability after GBNAT

**Parameters**		**Non-resectable**	**Resectable**	**Univariate**	**Multivariate**		
		**N = 24**	**N = 17**	***P***	***OR***	***95% CI***	***P***
Age	≥60y/o	18	12	0.735	0.939	0.876-1.00	0.075
	<60y/o	6	5				
CCRT	Yes	6	7	0.273			
	Nil	18	10				
Tumor location	Tail	0	6	**<0.001**	**50**	**1.218- >100**	**0.039**
	Head/body	24	11				
CEA(post-GBNAT)	<4.9	13	7	0.280			
	≥4.9	11	10				
CEA decrease (post-GBNAT)	Yes	10	8	0.821			
	Nil	14	9				
CA199 (post-GBNAT)	<152	9	13	**0.007**	**14.686**	**1.114-193.6**	**0.041**
	≥152	15	4				
CA199 decrease (post-GBNAT)	Yes	11	14	**0.008**	66.67	0.416- >100	0.105
	Nil	13	3				
CA125(post-GBNAT)	<32.8	12	9	0.790			
	≥32.8	12	8				
CA125 decrease (post-GBNAT)	Yes	14	4	**0.026**	8.547	0.138- >100	0.308
	Nil	10	13				

Bold letter means the p-values less than 0.05.

Table 3 showed the predictors for progression-free survival of LAPC patients following GBNAT. In univariate analysis, several factors were identified as predictors for progression-free survival, such as tumor location, pre-GBNAT CA-199 < 294, post-GBNAT CA-199 < 152, post-op CA-199 < 82, post-GBNAT CA125 < 32.8, and post-op CEA <6. However, after using multivariate analysis, **post-GBNAT CA-199 < 152 (OR 26.32, CI 3.300 ~ 200, P = 0.002) and post-GBNAT CA-125 < 32.8 (OR 55.56, CI 6.759 ~ 500, P < 0.001)** were significant predictors for patients with longer disease progression-free survival.

**Table 3:** Univariate and Multivariate analysis of risk factors for progression-free survival following GBNAT and surgical resection

**Parameter**	**Univariate**	**Multivariate**		
	***P***	**OR**	**95% CI**	***P***
Age	0.362	0.990	0.914-1.072	0.806
Tumor location: tail vs head or body	0.004	2.048	0.495-8.474	0.323
CA 199: pre-GBNAT <294 vs ≥294	0.003	1.776	0.357-8.850	0.483
CA 199: post-GBNAT <152 vs ≥152	0.000	**26.32**	**3.300-200**	**0.002**
CA 199: post-op <82 vs ≥82	0.007	2.137	0.524-8.696	0.290
CEA: post-op <6 vs ≥6	0.04	2.604	0.749-9.091	0.132
CA 125: post-GBNAT <32.8 vs ≥32.8	0.035	**55.56**	**6.579-500**	**<0.001**

*Abbreviations*: *OR* odds ratio, *95% CI* 95% confidence interval. Bold letter means the p-values less than 0.05.

The predictors for overall survival following GBNAT were shown in Table 4. Using univariate analysis, tumor location, resectable operation, post-op CEA < 6, pre-GBNAT CA-199 < 294, post-GBNAT CA-199 < 152, post-op CA-199 < 82, and post-GBNAT CA-125 < 32.8 were significance. Using multivariate analysis, **post-op CEA < 6 (OR 0.054, CI 0.005 ~ 0.0631, P = 0.020), pre-GBNAT CA-199 < 294 (0.033, CI 0.002 ~ 0.522, P = 0.015), and post-GBNAT CA-125 < 32.8 (OR = 0.034, CI 0.003 ~ 0.372, P = 0.006)** were significant predictors for patients with longer overall survival.

**Table 4:** Univarite and multivariate analysis of risk factors for overall survival following GBNAT and surgical resection

**Parameter**	**Univariate**	**Multivariate**		
	***P***	**OR**	**95% CI**	***P***
Age	0.308	0.982	0.891-1.082	0.709
Tumor location: head or body vs tail	0.003	4.689	0.048-458.2	0.509
Resectable operation: resectable vs non-resectable	0.001	5.492	0.122-246.7	0.380
CEA: post-op <6 vs >6	0.059	**0.054**	**0.005-0.631**	**0.020**
CA 199: pre-GBNAT <294 vs >294	0.011	**0.033**	**0.002-0.522**	**0.015**
CA 199: post-GBNAT <152 vs >152	0.008	0.464	0.041-5.672	0.536
CA 199: post-op <82 vs >82	0.000	0.201	0.015-2.652	0.223
CA125: post-GBNAT <32.8 vs >32.8	0.018	**0.034**	**0.003-0.372**	**0.006**

*Abbreviations*: *OR* odds ratio, *95% CI* 95% confidence interval. Bold letter means the p-values less than 0.05.

After GBNAT and surgical intervention, the metastatic/recurrent patterns were different in groups of patients with or without surgical exploration. Based on MDCT during the follow up period, 1/17 (6%) cases had loco-regional recurrence after surgical resection. The ratio of liver metastasis and peritoneal metastasis were improved in patients with surgical exploration compared to those without surgical exploration, 40% versus 100% and 30% versus 57.1%. However, the ratio of other distant metastasis was similar (Table 5).

**Table 5:** Patterns of failure after gemcitabine-based neoadjuvant therapy in locally advanced pancreatic cancer

**Metastatic/Recurrent Sites**	**Surgery n = 20 (%)**	**Non-surgery/n = 21 (%)**
**Liver**	8 (40%)	21 (100%)
**Peritoneum**	6 (30%)	12 (57.1%)
**Others (bone, lung, soft tissue, brain)**	5 (25%)	5 (23.8%)
Loco-regional recurrence in resectable cases*	1 (6%)	0
**Disease free**	3 (15%)	0

*One of the 17 resectable cases.

## Discussion

Surgery is the mainstay of treatment that offers significant survival in patients with pancreatic cancer, however, the overall survival is still poor due to low resectability. The challenging milestone for the improvement of outcome in LAPC is to increase the chance of surgical resection of patients either using chemotherapy or radiotherapy or combination [12-17]. Those patients who can benefit from neoadjuvant therapy and have the chance of surgical resection are still uncertain. In 2003, we set an algorithm for management of LAPC using GBNAT and responsive patients underwent surgical exploration at National Cheng Kung University Hospital. Following GBNAT, our study showed 17 of the 41 (41.5%) LAPC patients can be resected with a lower positive margin rate 17.6% (3 of 17 patients). Tumor location and post-GBNAT CA19-9 < 152 can be used as predictors for surgical resection. Post-GBNAT CA19-9 < 152 and post-GBNAT CA-125 < 32.8 are both predictors for longer disease progression-free survival. Patient with pre-GBNAT CA19-9 < 294, post-GBNAT CA-125 < 32.8 and post-op CEA < 6 had significant longer overall survival.

There were three major points of concern in the management of LAPC prior surgery. Firstly, what is the effective preoperative neoadjuvant regimen for LAPC? From the report of Gastrointestinal Tumor Study Group (GITSG), 5-fluorouracil (5-Fu) based chemoradiation can increase survival of pancreatic cancer patients [4]. Several studies used 5-Fu based chemoradiation to treat LAPC and the improvement of resection rate varies [4-6,18]. Kim HJ et al. found that in spite of the use of various chemoradiation protocols, it was impossible to downsize the tumor to obtain resectability and only one of 87 patients could be resected in that study [18]. However, Wanebo et al., using 5-Fu based chemoradiation, reported a resection rate up to 65% in 14 patients with LAPC [6]. Over the past 10 years, gemcitabine has become the standard of chemotherapy in advanced pancreatic cancer, and is also noted to be a potent radiosensitizer of epithelial cancer. Heinemann et al. reported that gemcitabine-based combination chemotherapy applied in advanced pancreatic cancer could show survival benefit, especially in those pancreatic cancer patients with a good performance status [8]. Many phase I and II studies demonstrated the feasibility of combining radiation with low dose gemcitabine weekly followed sequential full-dose gemcitabine [9]. These neoadjuvant treatments with gemcitabine-based CT or CRT was able to increase the resectability rate with clear margin and improved the prognosis of curative cases with comparable survival as initially resectable pancreatic cancer. Gillen et al. reported one-third unresectable tumor patients could be resected after neoadjunvant therapy [19]. A meta-analysis of 20 phase 3 trials by Bria E et al. concluded that gemcitabine-based chemotherapy could improve progression free survival in selected patients with inoperable pancreatic cancer [7]. In our series, 41.5% of LAPC patients could be resected; 55.6% (5 of 9) in previous failed exploration (borderline resectable) patients and 37.5% (12 of 32) in LAPC patient with long-term comparable outcome as initial resectable pancreatic cancer. Though different regimens were used in these patients, our results confirmed the efficacy of gemcitabine-based combination CT or CRT in our protocol.

The second concern is the definition of locally advanced pancreatic cancer. In the 6^th^ edition AJCC staging of pancreatic cancer, the pancreatic tumor with involvement of SMA or celiac plexus was rendered unresectable [2]. Occlusion of the confluence of portal vein (PV) and superior mesentery vein (SMV) was also considered unresectable according to the definition of resectable pancreatic cancer [11]. In 2006, a new category of borderline resectable pancreatic cancer was proposed by Varadhachary based on the extent of artery involvement and technical capability of reconstructing the vein [20]_._ The Fox Chase Cancer Center also suggested that tumor-induced unilateral shift or narrowing of the SPMV confluence as one of criteria of borderline resectable [21]. In resectable pancreatic cancer, the reported positive margin rate (R1 + R2) ranged from 19% to 68% and the positive margin strongly predicts the short survival and early recurrence rate [20]. Thus, patients with borderline resectable pancreatic head cancer are at higher risk for a margin-positive resection. The consensus of the Fox Chase group and the American-Hepato-Pancreato-Biliary Association (AHPBA)-Society of Surgical Oncology (SSO)-Society for Surgery of the Alimentary Tract (SSAT) suggested that borderline resectable pancreatic cancer should be treated with induction therapy before surgery [22]. In our study, the inclusion criteria were according to the 6^th^ edition of AJCC, tumor involving the confluence of portal vein and superior mesentery veins, tumor with severe extra-pancreatic soft tissue invasion, and previous failed exploration. Our recruited patients were 32 unresectable locally advanced pancreatic cancer and 9 patients with failed prior surgical exploration were borderline resectable pancreatic cancer. It is compatible with the definition of LAPC by 6^th^ edition AJCC staging of pancreatic cancer.

The third concern is the predictors for resectability and survival after GBNAT. The response of pancreatic cancer for GBNAT was based on the MDCT image findings. The criteria of exploration after GBNAT are downsizing of lesion and the clear plan between tumor and celiac artery/superior mesentery artery from the followed up MDCT [10]. Though the advancement in MDCT improved the accuracy of diagnosing tumor invasion in the area of SMA and celiac trunk, it is still unable to distinguish neoplastic reaction and fibrosis tissue, and can result in high unresectable rate even when considered as a resectable. Kim reported that neoadjuvant therapy could reduce the accuracy in tumor restaging [23], a possible reason for failure in exploration after neoadjuvant therapy. Massucco et al. reported that the interventions were more technically demanding [14] which reflect the difficulty in resection of LAPC after neoadjuvant therapy. Chao et al. reported that only minority of patients with unresectable tumor might become resectable after neoadjuvant treatment, and some of these resectable cases required portal venorrhaphy and hepatic artery reconstruction [13]. The absence of reliable biological or radiological predictor for exploration, a more aggressive policy, to explore all patients without disease progression, in order to improve the resection rate was suggested by Massucco et al. [14]. From our results, the surgical resection rate was 85% (17 of 20) and all the 3 failed re-explored patients were pancreatic head cancer with severe dense fibrosis between the retroperitoneal region and mesentery root area. The 6 tail patients could be resected after treatment because there was no hindrance in multi-organs resection for pancreatic surgeon. The multivariate analysis of clinicopathological factors showed that tumor location and post-GBNAT CA19-9 < 152 could be used as predictors for resection after GBNAT. Recently, based on this study, we have applied the predictors as criteria for exploration and SMA approach with portal vein reconstruction for uncinated process pancreatic cancer to increase resection rate. Now, the resected LAPC has increased to 30 cases in our hospital.

Surgery alone is not a good option for LAPC because of the high probability of incomplete surgical resection with residual cancer at the surgical margin or in draining lymph nodes. Multidisciplinary approach using CT or CRT is required to improve the survival rate of LAPC. Previous reports have showed that one-third of the selective borderline resectable pancreatic cancer or LAPC can achieve longer disease free survival [19]. In comparison, the positive lymph node and the positive margin rate was lower than the previous report and might be one of the reasons for better outcome in our study. Massucco et al. agreed that R0 resection give the chance of longer survival [14]. Takahashi et al. identified CA19-9 that substantially decreased after preoperative CRT was an indicator for therapeutic selection and survival [24]. In this study, we also found that patients with decreased preoperative CA19-9 and decreased preoperative CA125 were predictors for resectability in univariate analysis, but the significance vanished under multivariate analysis. However, our analysis proved that post-GBNAT CA-199 < 152 and post-GBNAT CA-125 < 32.8 were both predictors for patients with longer disease progression-free survival, and post-op CEA < 6, pre-GBNAT CA-199 < 294, and post-GBNAT CA-125 < 32.8 had significant longer overall survival.

## Conclusion

In conclusion, treatment of LAPC is challenging and requires multidisciplinary approach. With the advancement in neoadjuvant therapy and surgical techniques, we can improve the local and distant tumor control. Patients with resected LAPC following GBNAT can be expected to have comparable survival with initial resectable pancreatic cancer. Tumor location at pancreatic tail, post-GBNAT CA19-9 < 152, and post-GBNAT CA-125 < 32.8 could be used as predictors for resectability, disease-free survival, and overall survival of LAPC after GBNAT.

## Competing interests

The authors declare that they have no competing interests.

## Authors’ contributions

In this study, the surgery was performed by YJC and YSS, treatments were performed by YSS. YJC and EDS written the manuscript and revised the manuscript initially and final revision was done by YSS. The biostatistic was performed by HPH. All the study was performed under the supervision of YSS. All authors read and approved the final manuscript.

## Pre-publication history

The pre-publication history for this paper can be accessed here:

http://www.biomedcentral.com/1471-2482/14/72/prepub
